# Correspondence

**Published:** 1902-04

**Authors:** 


### Correspondence.

**For Texas Medical Journal.**

Died, February 19, 1902, at Marlin, Texas, after a few days' illness, Miss Lula M. Easter, daughter of Dr. and Mrs. W. E. Easter, of Hamburg, Ark. Miss Easter attended Bellwood School at Anchorage, Ky., and then graduated from the Peabody Normal School, Nashville, Tenn., with the degree of L. I.

In the fall of 1896, she accepted a position as teacher in the public school of Marlin, Texas, before she had attained her twentieth year. She held the same position at the time of her death.

In the meantime, she had taken a thorough course in kindergarten training in Chicago, and expected to make that her work in the future. She was peculiarly fitted for such work. Although of a very delicate physique, she possessed the strong mental and moral magnetism that drew closely to her all who knew her. To know her well, was to love her well. Sensitive, refined and intellectual, she responded to such traits in others, and shrank from anything that was not pure and true.

Miss Easter was a consistent member of the Presbyterian church.

A FRIEND.

---

98 Villegas Street,
HAVANA, CUBA, March 6, 1902.

*F. E. Daniel, M. D., Editor and Publisher of the Texas Medical Journal, Austin, Texas.*

SIR: With a great deal of interest and enthusiasm I have read in your journal the excellent article of Dr. J. P. Oliver, of Caldwell, Texas, about the *Culex fasciatus* mosquito not being the only means by which yellow fever is transmitted from one person to another. I also read with equal concern and fervor your editorial comments in the same January number of the TEXAS MEDICAL JOURNAL. I take pleasure, therefore, in sending to you herewith enclosed, for translation and publication, a humoristic article published today in the Spanish newspaper of this City, *El Nuevo País,* which is a terrible blow to that sophistry.

In the improperly called International Sanitary Congress (only three American nations having sent delegates to it) just held in this city of Havana, I was the only physician who had the courage to raise his voice at a public session against the adoption of the foolhardy and quarantine revolutionizing resolution that "the *Stegomyia fasciata* mosquito (and, mind you, the female ones alone, and after several days of an incubation period) is the only means by which yellow fever is transmitted from one person to another." My argumentation in reply was made in the presence of an imposing array of military opponents.

Dr. Joseph Y. Porter, State Health Officer of Florida, also opposed that resolution. However, he did not do so at a public session, but at a private meeting of the official delegates.

I have ready for publication a monograph which, if you so desire, I shall send also to your gallant and intrepid journal. It is entitled "The Mosquito Sanitary Fallacy."

Respectfully yours,
A. M. Fernandez Ibarra, A. B., M. D.,
Of New York City.

